# Comparing Race/Ethnicity and Zip Code Socioeconomic Status for Surgical versus Nonsurgical Management of Proximal Humerus Fractures in a Medicare Population

**DOI:** 10.5435/JAAOSGlobal-D-22-00205

**Published:** 2023-05-04

**Authors:** Peter Y. Joo, Christopher Wilhelm, Giscard Adeclat, Scott J. Halperin, Jay Moran, Ali Elaydi, Lee E. Rubin, Jonathan N. Grauer

**Affiliations:** From the Department of Orthopaedics and Rehabilitation, Yale School of Medicine, New Haven, CT.

## Abstract

This study evaluated the effect of race/ethnicity and socioeconomic status (SES) on surgical utilization after proximal humerus fractures in a large Medicare cohort.

**Methods:**

The PearlDiver Medicare claims database was used to identify patients aged 65years and older with isolated, closed proximal humerus fractures, for whom race/ethnicity data were available (65.5% of identified fractures). Patients with polytrauma or neoplasm were excluded. Patient demographic, race/ethnicity, comorbidity, and median household income were compared for surgical versus nonsurgical management. Univariate and multivariable logistic regressions were used to determine disparities of surgical utilization based on the abovementioned factors.

**Results:**

Of 133,218 patients with proximal humerus fracture identified, surgery was conducted for 4446 (3.3%). Those less likely to receive surgery were older (incrementally by increasing age bracket up to 85 years and older odds ratio [OR], 0.16, *P* < 0.001), male (OR, 0.79, *P* < 0.001), Black (OR, 0.51, *P* < 0.001) or Hispanic (0.61, *P* = 0.005), higher Elixhauser Comorbidity Index (per 2 increase OR, 0.86, *P* < 0.001), and low median household income (OR, 0.79, *P* < 0.001).

**Conclusions:**

The independent significance of race/ethnicity and SES point to disparities in surgical decision making/access to care. These findings highlight the need for increased attention on initiatives and policies that seek to eliminate racial disparities and improve health equity independent of SES.

Proximal humerus fractures are common injuries in the elderly Medicare population, with the frequency anticipated to increase with the aging population.^1-3^ Although a common injury, the management decisions for many isolated and closed proximal humerus fractures remain controversial.^4-6^ Most patients are treated nonsurgically^7^; however, surgical management has been increasingly pursued over the past few decades related to innovations in methods for stabilization and arthroplasty.^8-11^

Racial and ethnic disparities have been reported in surgical decision making for coronary artery bypass grafting,^12,13^ carotid endarterectomy,^12,13^ total hip arthroplasty,^12,13^ heart valve arthroplasty,^12^ and the general orthopaedic patient population.^14^ These studies have shown that healthcare decisions may be made differently for minority patients and underserved groups in comparison with non-Hispanic White patients. Although racial disparities for surgical utilization after proximal humerus fractures have not been elucidated in the literature, Khatib et al^15^ used a New York State database to demonstrate racial disparities in the utilization of shoulder arthroplasty in general.

In considering racial and ethnic disparities in care, controversy exists on whether other factors, such as socioeconomic status (SES), act as confounders.^16-18^ Rangrass et al and Skinner et al linked patient zip code to census-reported income in Medicare populations as proxies for median household income (MHI)/SES.^19-21^ They found that race/ethnicity and income were independent factors associated with receiving total knee arthroplasties and outcomes after cardiac surgery.

Overall, patient demographic and comorbidity factors, as well as fracture patterns, are known to affect surgical decision making for the management of proximal humerus fracture in the geriatric Medicare population. To better define these and other variables that affect this decision algorithm, this study sought to address the following questions:

1. What are the differences in patient characteristics between those who received surgical and nonsurgical management of isolated closed proximal humerus fractures in Medicare patients?
2. After controlling for patient demographics and comorbidity factors, do race/ethnicity and socioeconomic zip code status disparities exist in the management of isolated closed proximal humerus fractures?

## Patients/Methods

### Study Design and Setting

This study was a retrospective review of the 2014 to 2016 PearlDiver Standard Analytical Files Medicare claims database (PearlDiver Technologies), a large administrative data set containing records of over 51 million Medicare lives. PearlDiver Standard Analytical File includes all patient records billed to Medicare. Studies using the PearlDiver databases were exempted for ethical approval by the Institutional Review Board of Yale School of Medicine.

### Participants

Cases of closed proximal humerus fractures were identified using the relevant International Classification of Diseases-9 diagnosis codes (812.00 to 812.02 and 812.09) and International Classification of Diseases-10 diagnosis codes (S42.20XX to S42.24XX and S42.29XX). Exclusion criteria included age 65 years and younger, open fractures, other concurrent upper extremity fractures (to obtain a cohort of isolated proximal humerus fracture patients), and upper extremity neoplasm. Patients without race/ethnicity data were also excluded, but the subcohort with these data was notably compared with the entire cohort identified, as described under Study Population below.

Patients undergoing surgical intervention within 30 days after fracture were identified by the presence of current procedural terminology (CPT) codes for open reduction and internal fixation (CPT 23615) and arthroplasty (CPT 23616). These two surgeries were combined into one cohort to ensure adequate power for analysis. Those with closed proximal humerus fractures without these two CPT codes were assumed to have undergone closed management of the fracture. Mutual exclusion between the two cohorts was ensured.

Patient characteristics were then extracted, including age, sex, self-reported race/ethnicity (White, Black, Hispanic, Asian, Native American, and Other [mixed or other self-reported category not otherwise specified]), Elixhauser Comorbidity Index (ECI, a list of 31 comorbidity categories developed to measure a patient's general comorbidity burden), and estimated MHI by zip code. Race/ethnicity and zip code data were reported by Medicare, and patients could only be included in one race/ethnicity and zip code category. To obtain the estimated MHI, the zip code was linked to the MHI from the publicly available 2015 US Census MHI data. “High” MHI was classified as ≥$75,000, and “low” MHI was classified as <$75,000.

### Statistical Analysis

Univariate analysis was conducted to compare patient characteristics and proportion receiving either treatment option using the independent two-tailed Student *t*-test for continuous variables and the chi square test for categorical variables. For race/ethnicity, the proportional ratios of treatment options within each category were compared and visualized.

To determine the independent relationship of race/ethnicity to surgical management of proximal humerus fractures, multivariable logistic regression analysis was then conducted, controlling for the variables assessed. Odds ratios (OR) in obtaining surgical treatment versus nonsurgical management and 95% confidence intervals were reported.

An alpha of 0.05 and significance of *P* < 0.05 were set for both the univariate and multivariable analyses. Statistical calculations were conducted in PearlDiver software using the R statistical language. Figures were created with Excel (Microsoft Corporation).

## Results

In total, 203,368 patients with any closed proximal humerus fractures met the inclusion criteria of isolated fracture in patients 65 years and older. Of these patients, 133,218 patients (65.5%) were with race/ethnicity identifiers.

To assess whether there was a selection bias for the reporting of race/ethnicity data, patients with these data were compared with those without. Variability for age ranges was within 1% in each category, and while statistically significant because of the power, this was thought to be clinically comparable. Sex, ECI, and distribution of high income by zip code were not found to be statistically different.

In the analysis, 133,218 patients who underwent surgical (n = 4,446, 3.3%) and nonsurgical (n = 128,772, 96.6%) management of isolated proximal humerus fractures were included. Among those who received surgical management, 3203 patients (72%) underwent open reduction and internal fixation, and 1243 patients (28%) underwent arthroplasty. Patient characteristics for each cohort are summarized in Table 1.

**Table 1:** Patient Characteristics by Closed Proximal Humerus Fracture Treatment Type

	Surgical	% (SD)	Nonsurgical	% (SD)	*P* Value
Total (n)	4446		128,772		
Age (yr)					**<0.001**
65–69	1533	34.5%	22,886	17.8%	
70–74	1177	26.5%	22,625	17.6%	
75–79	832	18.7%	22,178	17.2%	
80–84	540	12.1%	22,422	17.4%	
≥85	364	8.2%	38,661	30.0%	
Sex					**<0.001**
Female	3736	84.0%	103,261	80.2%	
Male	710	16.0%	25,511	19.8%	
Race/ethnicity					**<0.001**
White	4252	95.6%	119,944	93.1%	
Black	71	1.6%	3923	3.0%	
Hispanic	33	0.7%	1732	1.3%	
Asian	34	0.8%	1266	1.0%	
Native American	28	0.6%	676	0.5%	
Other	28	0.6%	1231	1.0%	
ECI	5.1	(3.8)	7.1	(4.4)	**<0.001**
MHI by zip code					**<0.001**
High—≥$75,000	1272	28.6%	30,759	23.9%	
Low—<$75,000	3174	71.4%	98,013	76.1%	

ECI = Elixhauser Comorbidity Index, MHI = median household income, SD = standard deviation

Boldface = significant at *P* < 0.05.

**Figure 1:**
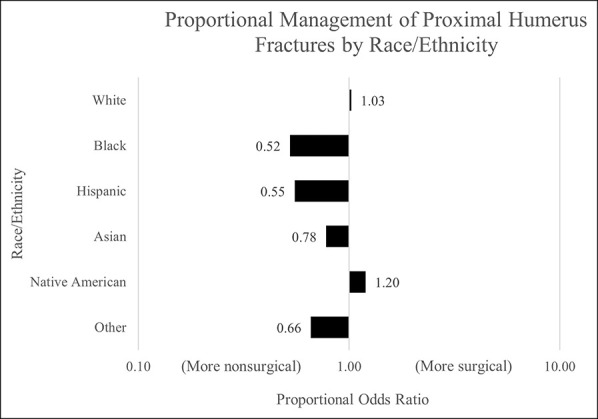
Graph showing proportional odds of surgical management of proximal humerus fractures, stratified by race/ethnicity. Overall differences in proportional chi square odds are noted to be statistically significant (*P* < 0.001).

The overwhelming majority of patients with isolated closed proximal humerus fractures were treated nonsurgically (96.7%), and these patients were more likely to be older, be male, and have greater ECI than those treated surgically (*P* < 0.001 each) (Table 1). There were also differences by race/ethnicity: numerically presented and compared by using the chi square test, as presented in Table 1, and by univariate proportional ratio analysis, as shown in Figure 1 (*P* < 0.001). Patients who identified as Black, Hispanic, Asian, and Other had lower proportional odds of surgical management of proximal humerus fractures, whereas patients who identified as White or Native American had higher proportional odds of surgical management. Finally, those with lower MHI by zip code were also more likely to receive nonsurgical management (*P* < 0.001) (Table 1).

Multivariable logistic regressions controlling for the variables assessed are summarized in Table 2. The OR of surgical management decreased with incrementally increasing age, from 70 to 74 years (OR, 0.85) to 85 years and older (OR, 0.16), *P* < 0.001, compared with the age group of 65 to 69 years. Male patients were less likely to undergo surgery than female patients (OR, 0.79, *P* < 0.001). When compared with White patients, several groups were less likely to undergo surgery (Black: OR, 0.51, *P* < 0.001; Hispanic: OR, 0.61, *P* = 0.005; and Other: OR, 0.58, *P* = 0.003). Patients with greater comorbidity were less likely to undergo surgery (OR, 0.86, *P* < 0.001, per ECI increase of 2), and those with lower MHI by zip code were less likely to undergo surgery (OR, 0.79, *P* < 0.001). All these odds were independent of the other included variables, showing that racial disparities persist even after controlling for demographic, comorbidity, and SES factors.

**Table 2:** Independent Predictors of Surgical Treatment of Proximal Humerus Fractures

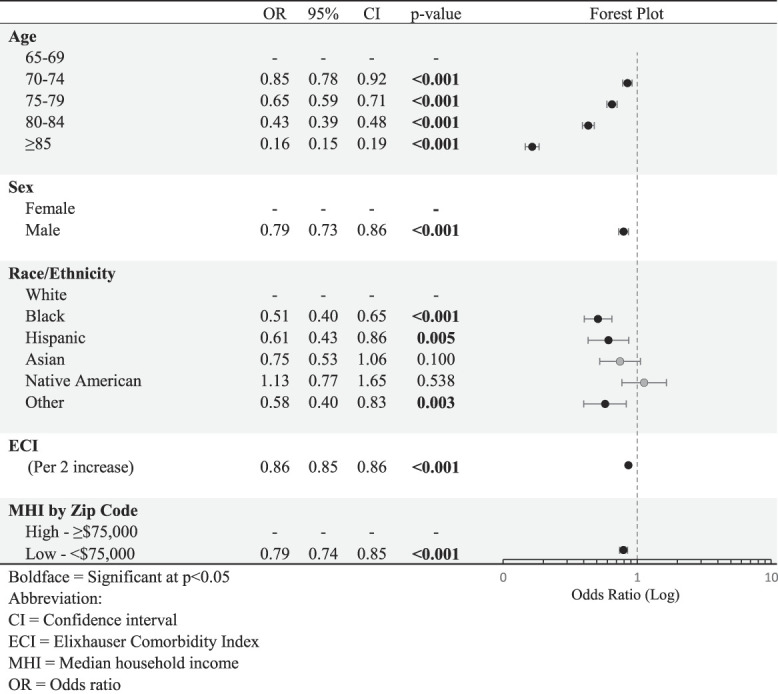

## Discussion

This is the first study to assess racial disparities in surgical utilization for proximal humerus fractures in the Medicare population, controlling for SES and other patient factors. Disparities in surgical utilization may be due to multiple factors, such as racial bias, SES, hospital/surgeon characteristics, and more, and the relative effects of each variable on disparities remain widely debated in the surgical literature.^12-14,16-18,20,22^ Thus, it is ever more important to better elucidate independent effects of race/ethnicity on how surgeons treat and manage their patients.

On univariate analysis, higher estimated MHI by zip code and White female patients were markedly associated with higher surgical management of proximal humerus fractures. Those who received proportionally less surgical management included Black, Hispanic, Asian, and Other patients. Although no previous studies have evaluated socioeconomic and racial differences in utilization of surgical management after proximal humerus fractures, the other patient characteristics are in line with previous studies.^2,3,7,10,11,23,24^ The closest study by Khatib et al^15^ demonstrated that racial disparities did exist for the overall utilization of shoulder arthroplasty for all indications in the state of New York, with White patients representing approximately 80% of arthroplasty cases and Black patients representing less than 5%.

On multivariable analysis, race/ethnicity was found to be independently associated with surgical utilization even after controlling for age, sex, comorbidities, and SES. Black, Hispanic, and Other patients had markedly lower odds of surgical management compared with White patients. While those living in higher SES zip codes did independently receive more surgical intervention, this effect did not markedly affect the effect of race/ethnicity. This is in line with two similar orthopaedic studies by Skinner et al^21^ and Neuman et al,^22^ where they found notable racial disparities in surgical utilization for total knee arthroplasty and geriatric hip fractures in the Medicare population, respectively, that did not reduce markedly after controlling for SES factors.^21,22^

This study does have limitations. As with other administrative database studies, it is reliant on the accuracy of administrative coding and the degree and fracture-specific details, such as fracture pattern and number of parts, cannot be assessed with used ICD coding. Furthermore, race/ethnicity data were only present for a subset of the patients, but these patients seemed to be clinically similar to those without this variable. Although a multivariable regression was used to identify independent effects of each variable and reduce confounding variables, unfortunately, it is not possible to eliminate all potential confounders. The benefit of database studies is that large sample sizes will tend toward the mean and reduce variability, allowing for leniency in controlling for certain variables. Despite these limitations, the large cohort of over 133,218 patients assessed in this study have not been otherwise accessible in previous studies, and this is the first study on proximal humerus fractures to control for variables such as SES by zip code. Future studies using updated databases and prospective studies can look to examine further the race/ethnicity and SES disparities in surgical decision making and identify how surgical utilization and decision making are associated with clinical outcomes.

## Conclusion

Although various factors are known to affect surgical decision making for proximal humerus fractures, such as age and comorbidity burden, others such as SES and race/ethnicity have not been previously reported and point to a disparity in surgical decision making/access to care. These findings highlight the need for increased attention on initiatives and policies that seek to eliminate racial disparities and improve health equity independent of SES.
